# Analogous reserve distribution and tissue characteristics in quinoa and grass seeds suggest convergent evolution

**DOI:** 10.3389/fpls.2014.00546

**Published:** 2014-10-16

**Authors:** Hernán P. Burrieza, María P. López-Fernández, Sara Maldonado

**Affiliations:** ^1^Instituto de Biodiversidad y Biologia Experimental y Aplicada – Consejo Nacional de Investigaciones Científicas y Técnicas, Ciudad Autónoma de Buenos AiresArgentina; ^2^Departamento de Biodiversidad y Biología Experimental, Facultad de Ciencias Exactas y Naturales, Universidad de Buenos Aires, Ciudad Autónoma de Buenos AiresArgentina

**Keywords:** coleorhiza, endosperm, grass seed, micropylar endosperm, perisperm, quinoa seed

## Abstract

Quinoa seeds are highly nutritious due to the quality of their proteins and lipids and the wide range of minerals and vitamins they store. Three compartments can be distinguished within the mature seed: embryo, endosperm, and perisperm. The distribution of main storage reserves is clearly different in those areas: the embryo and endosperm store proteins, lipids, and minerals, and the perisperm stores starch. Tissues equivalent (but not homologous) to those found in grasses can be identified in quinoa, suggesting the effectiveness of this seed reserve distribution strategy; as in cells of grass starchy endosperm, the cells of the quinoa perisperm endoreduplicate, increase in size, synthesize starch, and die during development. In addition, both systems present an extra-embryonic tissue that stores proteins, lipids and minerals: in gramineae, the aleurone layer(s) of the endosperm; in quinoa, the micropylar endosperm; in both cases, the tissues are living. Moreover, the quinoa micropylar endosperm and the coleorhiza in grasses play similar roles, protecting the root in the quiescent seed and controlling dormancy during germination. This investigation is just the beginning of a broader and comparative study of the development of quinoa and grass seeds. Several questions arise from this study, such as: how are synthesis and activation of seed proteins and enzymes regulated during development and germination, what are the genes involved in these processes, and lastly, what is the genetic foundation justifying the analogy to grasses.

## INTRODUCTION

Cereals, e.g., rice (*Oryza sativa* L.), wheat (*Triticum aestivum* L.), maize (*Zea mays* L.), barley (*Hordeum vulgare* L.), oat (*Avena sativa* L.), rye (*Secale cereale* L.) are members of the monocot family Poaceae, which are cultivated for the edible components of their grains or cariopses and consist of a single seed enclosed by dry and indehiscent pericarp, firmly adhered to the rest of the integuments.

**FIGURE 1:**
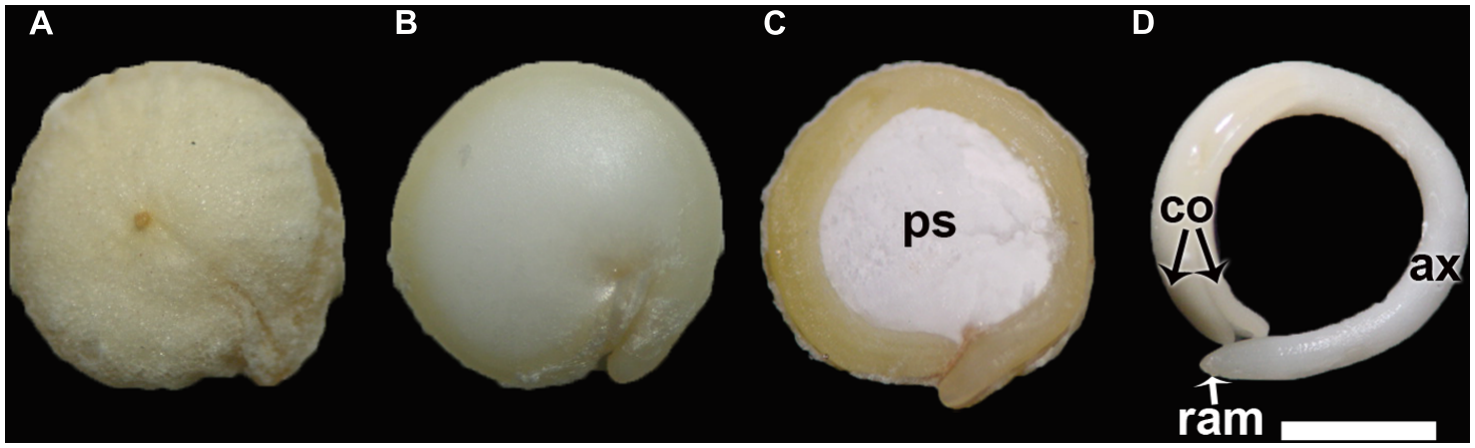
**(A)** Quinoa grain; **(B)** Quinoa seed (without pericarp); **(C)** Longitudinal midsection of a quinoa seed; **(D)** Excised embryo. *ax*, hypocotyl-radicle axis; *co*, cotyledon; *ps*, perisperm; ram, root apical meristem. Bar: 1 mm.

**FIGURE 2:**
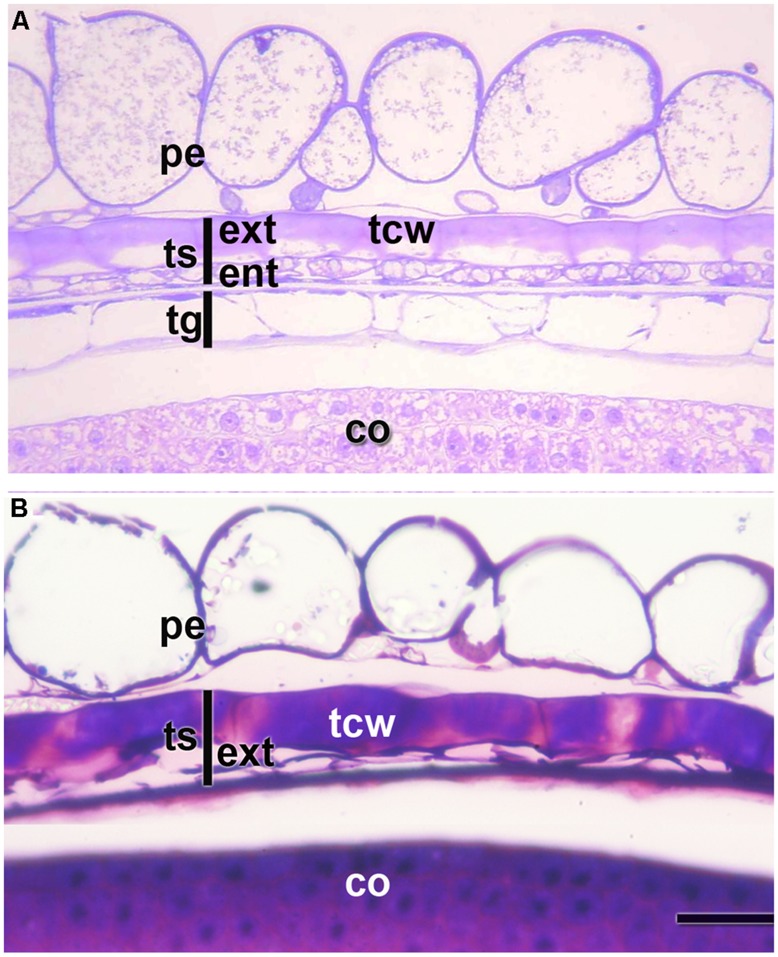
**Pericarp and integuments at two stages of quinoa seed development. (A)** Young seed (torpedo stage). **(B)** Mature seed. Grains were fixed using a mixture of 2% paraformaldehyde and 1% glutaraldehyde in 0.1 M phosphate buffer, pH 7.2, and embedded in London Resin White resin, according to López-Fernández and Maldonado (b). Semithin sections (2 μm thick) were stained with 0.5 % Toluidine Blue. *co*, cotyledon; *ent*, endotesta; *ext,* exotesta; *pe*, pericarp; *tcw*, tangential cell wall of the cells from the outer layer of the outer integument; *tg*, tegmen; *ts*, testa.

Pseudocereals are dicots (thus not cereals) and include species of the Amaranthaceae (e.g., quinoa, *Chenopodium quinoa* Willd., and different species of the genus *Amaranthus*) and Polygonaceae (e.g., buckwheat, *Fagopyrum esculentum* Moench) families, also cultivated for the edible components of their grains. The dispersal unit in pseudocereals is the grain botanically called achene, which consists of a single seed enclosed in a dry and indehiscent pericarp (**Figures 1** and **2**). According to Prego et al. (1998), in quinoa the pericarp is very thin; as a result, the achene is also referred to as utricle.

**FIGURE 3:**
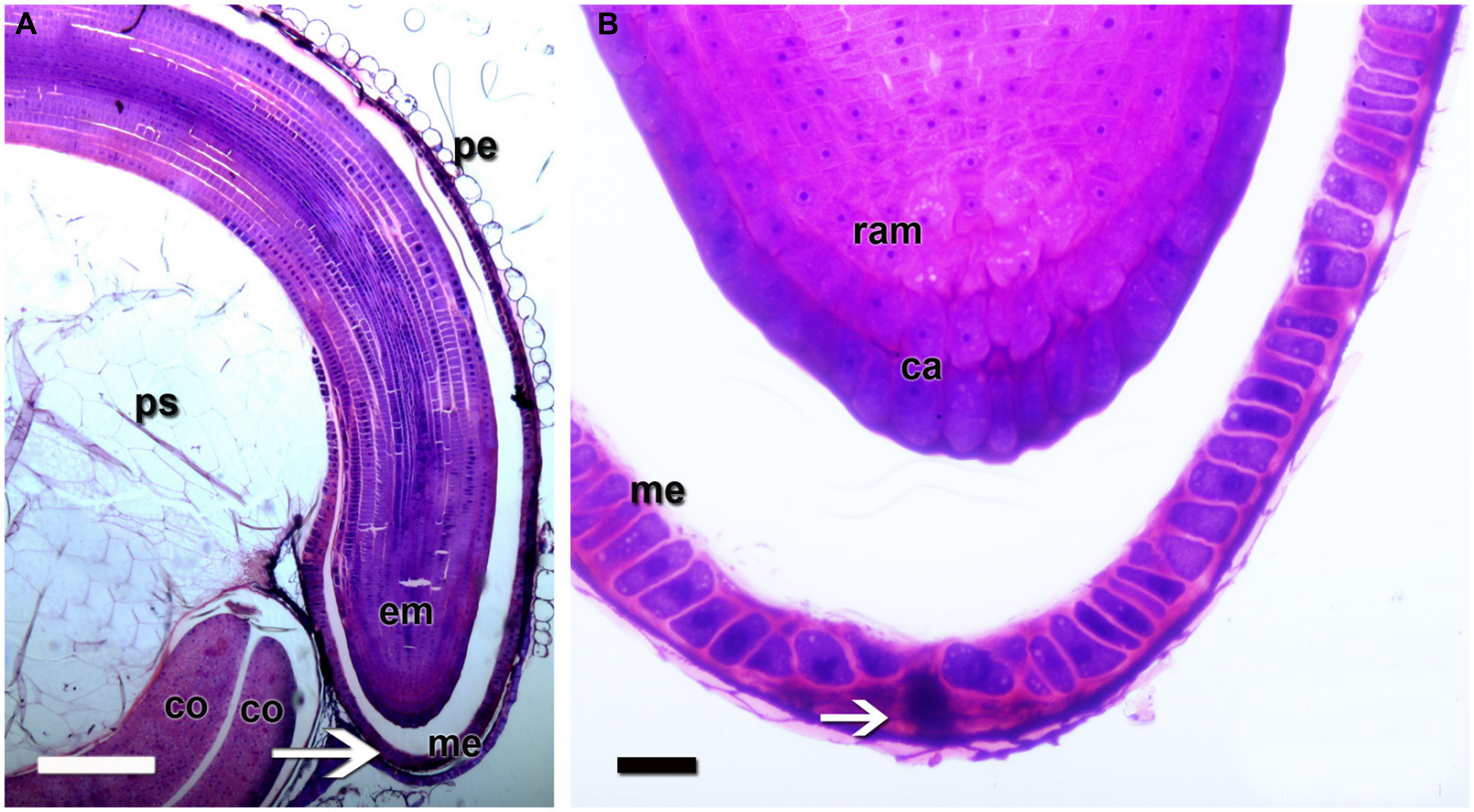
**Quinoa micropylar endosperm (in a mature seed). (A)** The white arrow indicates the center of the micropylar cone; *co*, cotyledon; *em*, embryo; *me*, micropylar endosperm; *pe*, pericarp; *ps*, perisperm. **(B)** Detail of **(A)**. The white arrow indicates the central channel of the micropylar cone, which is occupied by the remains of the suspensor. *ca*, caliptra; *me*, micropylar endosperm; *ram*, root apical meristem. Semithin section (2 μm thick) was obtained from a fixed, resin-embedded, sectioned, and stained seed, as described in **Figure 2**.

Quinoa seeds are highly nutritious due to the quality of their proteins and lipids and the wide range of minerals and vitamins they store. The ability of quinoa to produce high-quality proteins under extreme environmental conditions makes it an important crop not only for Andean communities but also for the diversification of future agricultural systems. Cereals and quinoa are grain crops and thus essentially full of starch, but they also contain significant quantities of proteins, oil, and minerals. In quinoa and grasses, the distribution of main storage reserves is clearly divided and three main storage compartments can be distinguished in the mature seed: (i) a tissue that stores mainly starch, which is the perisperm in quinoa and the starchy endosperm in grasses, with (ii) an embryo that stores principally proteins and lipids, and (iii) a non-embryonic tissue that stores proteins, lipids, and minerals, which is the aleurone layer in gramineae and the micropylar cone in quinoa. In this study we demonstrate that tissues equivalent (but not homologous) to those found in grasses can be identified in quinoa (**Figure 3**), thus suggesting the effectiveness of this seed reserve compounds distribution strategy and justifying the advantages that led evolutionary distant plants to develop seeds with analogous nutrients distribution.

This investigation is part of a broad comparative study of the development of quinoa and grass seeds. Several questions arise from this investigation, such as: how are synthesis of seed proteins and enzymes regulated during development and germination, what are the genes involved in these processes, and lastly, what is the genetic foundation for the analogy with grasses.

## THE FRUIT

According to Prego et al. (1998), in quinoa the pericarp is made up of papillose cells derived from the outer epidermis of the ovary and an inner discontinuous layer with tangentially stretched cells (**Figure 2**). The seed coat derives from ovule integuments, each one constituted by two to three layers. The seed coat consists of a testa and a tegmen, each one two layers thick. During seed development, the endotesta layer and both layers of the tegmen are almost completely consumed (**Figure 2B**); on the contrary, cells of the exotesta enlarge and develop thick tangential cell walls; these cells remain intact and are dismantled just after germination (**Figure 2**).

In grasses, the pericarp is adhered to the remains derived from the ovule integuments, which are, at maturity, usually reduced to a thin layer (Morrison, 1976). The anatomy of the pericarp is remarkably similar in all the grasses studied to date (Rost, 1973). It is composed of an outer epidermis with a thick cuticle layer and one to several subjacent cell layers (Bessey, 1894; Narayanaswami, 1953, 1955a,b,c, 1956; Rost, 1973). Even though cells of the subjacent layers are crushed, two types of cell layers, both typically containing cells with thick walls can be identified: a cross-cell layer, adjacent to the inner epidermis, whose cells are elongated transversely to the long axis of the caryopsis; and a tube cell layer, derived from the inner epidermis, whose cells are elongated parallel to the caryopsis axis (Krauss, 1933; Kent and Evers, 1994).

## THE SEED

### THE QUINOA SEED

The ovule is amphitropous (i.e., ovule is bent by the formation of a basal body, then both micropylar, and chalazal ends are near each other), bitegmic (with two integuments), and crassinucellate (archesporial cell cuts off a parietal cell, and parietal cell derivatives make the megasporocyte deep-seated in the ovule; Davis, 1966). The seed contains a peripheral, curved embryo surrounding a perisperm or basal body and is covered by integuments and pericarp (**Figure 1**). A micropylar endosperm forming a cone surrounds the root apical meristem of the embryo (**Figure 3**). The embryo consists of a hypocotyl–radicle axis and two cotyledons (**Figure 1D**). In the axis, both the root apical meristem, with the root cap, and the shoot apical meristem are differentiated. The shoot apical meristem forms a conical structure between the two cotyledons lacking leaf primordia (Prego et al., 1998; Burrieza et al., 2012). All embryo cells, including those of the apical meristems, store abundant proteins and lipids in the form of protein and lipid bodies, respectively. During germination, the radicle grows through the center of the cone, in the space previously occupied by the suspensor.

**FIGURE 4:**
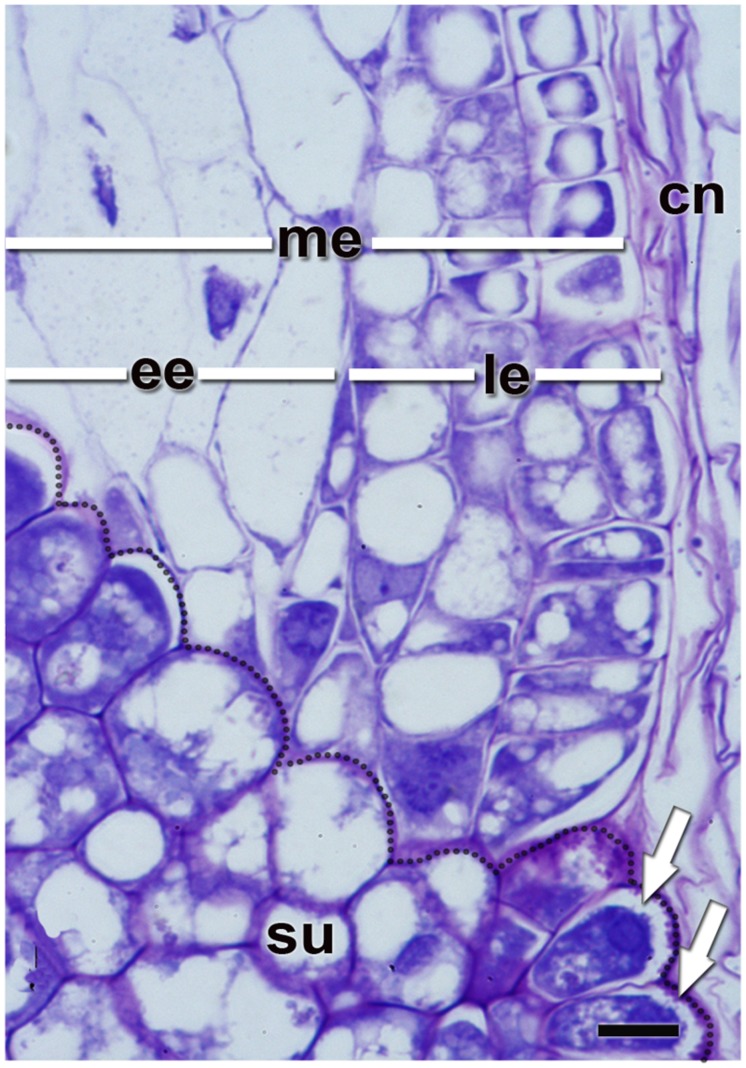
**Quinoa micropylar endosperm (at the torpedo stage).** *cn*, crushed nucellus; *ee*, ephemeral endosperm; *le*, lasting endosperm; *me*, micropylar endosperm; *su*, supensor. Semithin section (2 μm thick) was obtained from a fixed, resin-embedded, sectioned, and stained seed as described in **Figure 2**. The border between the suspensor and endosperm has been highlighted with a dotted line. White arrows indicate the “festooned” outer cell walls in the transfer cells of the suspensor. Bar: 10 μm.

**FIGURE 5:**
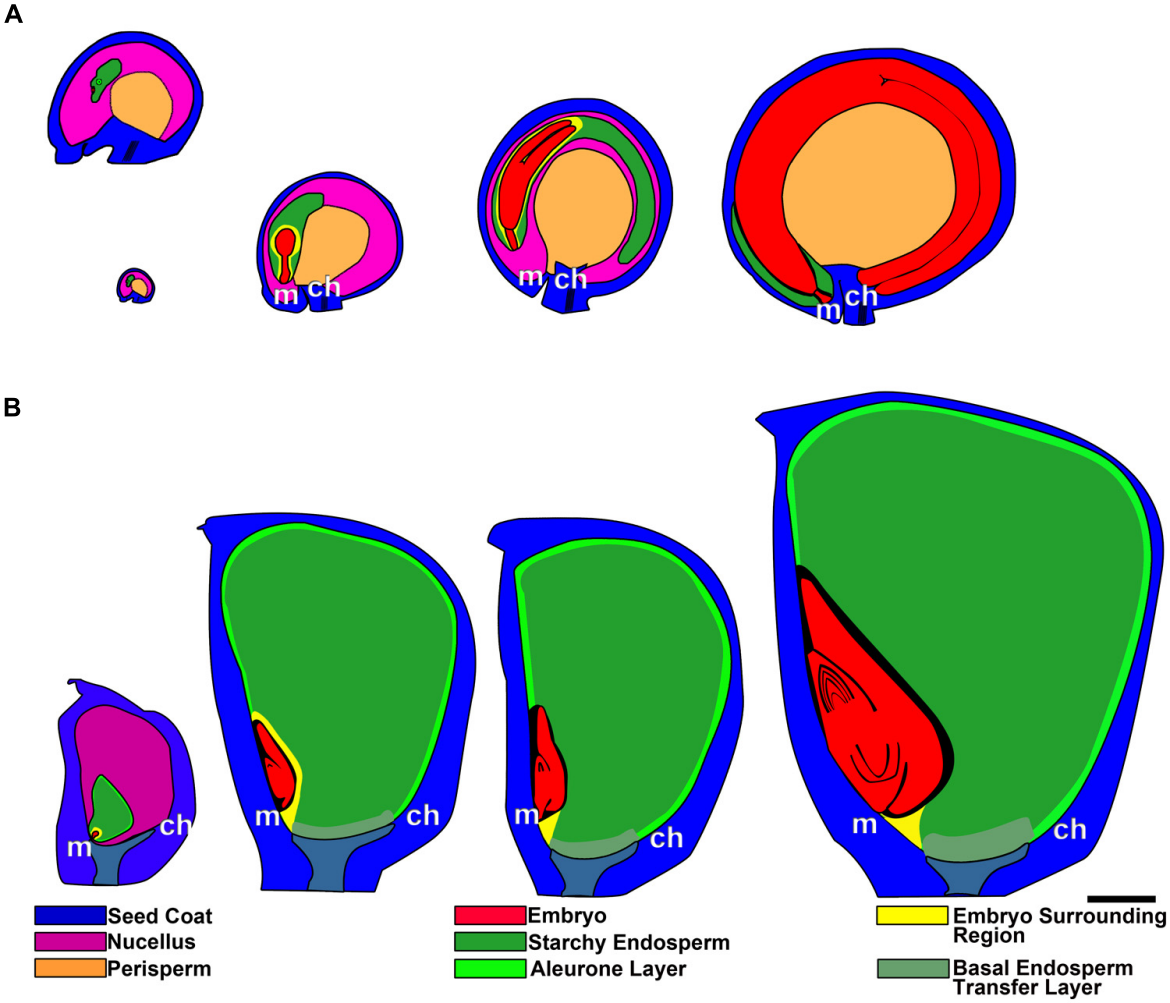
**Grain development in quinoa **(A)** and maize **(B)**, from anthesis to maturity.** In quinoa, the first image (ovule at anthesis) has been enlarged in the top image. *ch*, chalazal pole; *m*, micropylar pole. Bar: 0.5 mm.

The suspensor connects the embryo to the nucellus during early seed development, holding the growing embryo in a fixed position within the seed and allowing nutrients to be transported to the embryo (Bozhkov et al., 2005). It is a short-lived tissue, only active in early embryogenesis. In quinoa, it is constituted by (1) a neck that connects the suspensor to the embryo proper, which is made up of cells with small vacuoles; and (2) a knob, which is composed of a set of larger basal cells that protrude into the micropylar endosperm (**Figures 4** and **5**). The knob is formed by transfer cells on the outside, with ingrowths in their outer cell walls (**Figure 4**), dense cytoplasm, and numerous small vacuoles (López-Fernández and Maldonado, 2013b). When the embryo finishes accumulating reserves its cells degenerate leaving, however, visible remains in the center of the micropylar cone (**Figure 3**). During germination, this remnant of the suspensor presumably offers less mechanical resistance and might help facilitate radicle protrusion during germination.

The perisperm is derived from the nucellus of the ovule; this portion of the nucellar tissue is not consumed by the development of the embryo sac and persists after fertilization, becoming the main nutritive tissue of the seed (**Figure 5A**). During quinoa perisperm development, three major developmental phases can be distinguished: (1) early development of the nucellus, including mitotic activity, the last stage of which takes place before anthesis, establishing the final cell number and tissue configuration; (2) cellular differentiation, which can be broken down into the partly overlapping processes of cellular expansion, endoreduplication, accumulation of starch reserves, and programmed cell death (PCD); and (3) maturation, which comprises the shutdown of biosynthetic processes, desiccation induction, and quiescence (López-Fernández and Maldonado, 2013a). Through endoreduplication, DNA content usually peaks at 8°C but some nuclei can reach up to 16 and 32°C (López-Fernández and Maldonado, 2013a). At maturity, the perisperm consists of uniform, non-living, thin-walled cells. Nuclei and other cytoplasmic organelles are absent at this stage. Cells are full of compound starch grains and simple starch grains occupy the space between compound grains (Prego et al., 1998).

Endosperm development in quinoa, as in cereals, is of the nuclear type. According to Olsen (2004), syncytial and cellularization phases of nuclear endosperm development are conserved among all groups of angiosperms. Endosperm development has only recently begun to be studied in quinoa (López-Fernández and Maldonado, 2013b). In the endosperm mother cell, nuclear divisions occur freely in the parietal cytoplasm surrounding the central vacuole. Cellularization is associated with the initiation of periclinal divisions, which occur in a centripetal direction, thereby shrinking the vacuole. While divisions are ongoing, nuclei grow in size and endoreduplicate, with the DNA content peaking at 6°C (López-Fernández and Maldonado, 2013b). When the endosperm reaches its final size, three domains are differentiated: a micropylar domain, comprising six to eight cell layers; a peripheral domain of two cell layers; and a chalazal domain of six or seven cell layers. The embryo grows at the expense of the endosperm, i.e., the chalazal and peripheral endosperms, as well as the inner layers of the micropylar endosperm are progressively dismantled (**Figure 5A**). Cells destined to be consumed during embryo development do not accumulate storage reserves. In mature seeds, the remaining endosperm forms a micropylar cone, crossed in the center by the suspensor (**Figure 4**). According to López-Fernández and Maldonado (2013b), throughout development, two major cell types make up the quinoa endosperm: the micropylar endosperm, which is constituted by a cone covering the radicle, and the ephemeral endosperm: the tissue located on either side of the growing embryo (**Figure 4**). During seed development, cells of the ephemeral endosperm are crushed and finally disintegrated. In contrast, cells of the micropylar cone (**Figure 3**), which is one or two cell layers thick, store lipids and proteins that are used by the embryo during germination.

### THE GRASS SEED

The ovule is hemianatropous (the hemianatropous condition results from the curvature of the ovule such that the micropyle comes to lie at right angles to the funiculus), bitegmic (with two integuments), and tenuinucellate (i.e., the archesporial cell enlarges to form the megasporocyte, then megasporocyte is subdermal; Davis, 1966). The seed contains the embryo on the adaxial face of the caryopsis, which is unilaterally covered by the endosperm (**Figure 5B**).

The endosperm is almost completely preserved as reserve tissue in mature seeds. According to Olsen (2004), throughout development, four major cell types constitute the grass endosperm, i.e., aleurone, starchy endosperm, basal endosperm transfer cells, and embryo-surrounding region (**Figure 5B**). Similar to quinoa endosperm, after fertilization, the initial endosperm nucleus divides repeatedly without cell wall formation in the parietal cytoplasm surrounding the central vacuole (Brown et al., 1994; Olsen, 2004).

Cellularization is associated with the initiation of periclinal divisions, which occur in a centripetal direction, progressively shrinking the vacuole until its disappearance (Brown et al., 1994, 1996; Olsen, 2001, 2004). The first periclinal division round is formative, originating both the aleurone and starchy initials (Brown et al., 1996).

Aleurone cells form a sheet generally composed of cells that store proteins, lipids, and mineral nutrients to be used up during germination (Jones, 1969a; Lonsdale et al., 1999). In some species, the peripheral layer undergoes rounds of periclinal divisions before assuming aleurone cell characteristics. Thus, the amount of aleurone layers varies according to the species, e.g., maize and wheat have one layer, rice has one to several layers, and barley has three layers (Buttrose, 1963; Hoshikawa, 1993). Aleurone cells accumulate proteins and lipids in protein vacuoles and lipid bodies, respectively. Upon seed germination, it is assumed that storage proteins provides the amino acids necessary for the synthesis of hydrolytic enzymes required for starch mobilizing in the starchy endosperm and that aleurone cells die by autophagy (Jones, 1969b,c; Jones and Price, 1970; Taiz and Jones, 1970; Taiz and Honigman, 1976; for reviews see Bethke et al., 1998; Becraft and Gibum, 2011).

At maturity the starchy endosperm cells consist of uniform, non-living, thin-walled cells full of starch grains but they also contain protein bodies (Reyes et al., 2011; for review see Olsen et al., 1992). During development, these cells simultaneously accumulate storage reserves and degenerate, both processes mediated by a program of developmentally controlled cell death. Starchy endosperm cells accumulate starch in plastids and prolamins in protein bodies.

Endoreduplication occurs in starchy endosperm. In maize, DNA content usually peaks at 6 and 12°C, but some nuclei reach 24, 48, 96, and 192°C (Kowles and Phillips, 1985; Kowles et al., 1990; Larkins et al., 2001; Sabelli and Larkins, 2009; Sabelli, 2012). Endoreduplication precedes and accompanies two programs that occur simultaneously in this tissue: (i) accumulation of storage reserves, and (ii) PCD (Young et al., 1997; Young and Gallie, 2000; Domínguez and Cejudo, 2014).

The region of the transfer cells is located in the basal endosperm close to the vascular tissues of the placenta (**Figure 5B**), facilitating solute transfer from the vascular bundle of the pedicel toward the endosperm (Thompson et al., 2001). Two or three cells derived from the outer layer during the process of cellularization assume transfer cell identity, developing cell wall ingrowths (Thompson et al., 2001).

The embryo-surrounding region comprises several cell layers that completely envelop the young embryo (**Figure 5B**) and are characterized by their dense cytoplasm contents, abundance of small vacuoles, and a complex membrane system but they do not accumulate storage reserves and their death generates a space filled with crushed endosperm cells surrounding the embryo (Olsen, 2004; Sabelli and Larkins, 2009). The embryo grows at the expense of these cells, causing their degradation.

The single cotyledon of grasses is transformed into the absorptive scutellum which lies between the endosperm and the embryo axis. Many grasses possess a small scale-like appendage opposite the scutellum, the so-called epilates (Saha, 1957). The shoot apical meristem is covered by the coleoptile and contains several leaf primordia.

**FIGURE 6:**
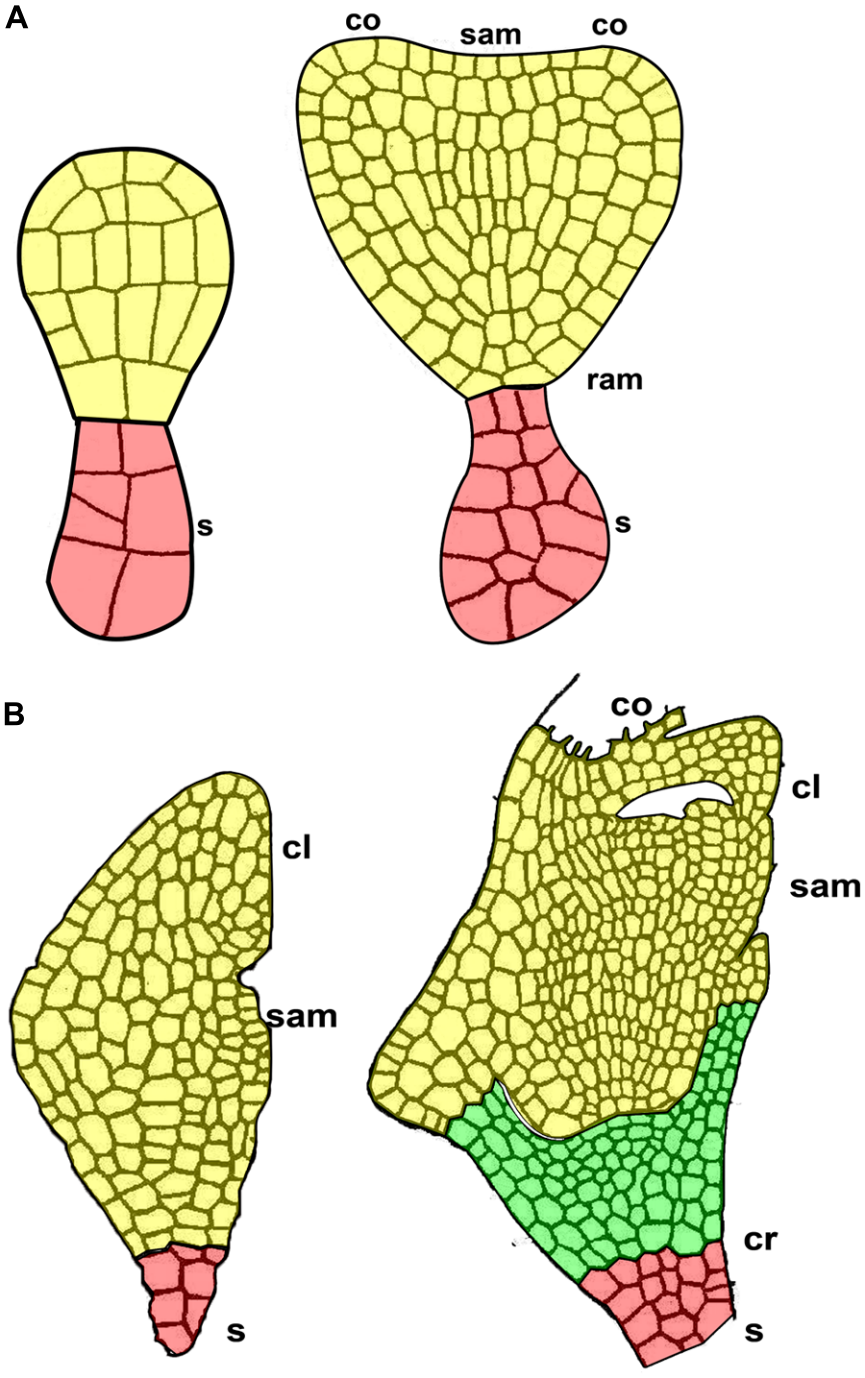
**The suspensor in two subsequent early stages of embryo development. (A)** Quinoa seed. **(B)** Grass seed. *cl*, coleoptile; *co*, cotyledon primordium; *cr*, coleorhiza; *ram*, root apical meristem; *sam*, shoot apical meristem; *s*, suspensor.

The coleorhiza is a non-vascularized, multicellular embryonic tissue that covers the cereal root apical meristem. It is an embryonic tissue located between the root apical meristem and the suspensor, characteristic of the grass embryo and absent in quinoa embryo (**Figure 6**). The coleorhiza originates together with the root cap and the suspensor (Johansen, 1950), but later in development it is separated from the radicle by a cleft (**Figure 6B**).

Although coleorhiza is involved in germination and successful establishment of all grass seedlings, information on its morphology, anatomy, and function is very sparse. In rye, Sargent and Osborne (1980) describe coleorhiza in the mature rye seed as made up of parenchymatic quiescent cells lacking vacuoles and containing a cytoplasm densely packed with ribosomes, lipid bodies largely confined to a peripheral position, a greatly reduced endomembrane system, mitochondria with a few cristae, and nuclei in which the heterochromatin is condensed. During germination of rye (Sargent and Osborne, 1980) and barley (Barrero et al., 2009) seeds, coleorhiza cells elongate and separate from each other, thereby forming intercellular spaces after consuming their own storage reserves. Eventually, the growth of embryonic roots (primary and adventitious) dismantles the tissue (Millar et al., 2006).

### MICROPYLAR ENDOSPERM vs. COLEORHIZA

In quinoa, the micropylar endosperm is the only part of the tissue that persists in the mature seed, forming a cone covering the root apical meristem (**Figure 5A**). In other species (e.g., tomato, *Datura ferox*), the micropylar endosperm is just the micropylar portion of a larger tissue covering the root apical meristem. The micropylar endosperm has been studied in the species *Arabidopsis thaliana* (Millar et al., 2006; Okamoto et al., 2006), cress (Müller et al., 2006), *Datura ferox* (Mella et al., 1995; Arana et al., 2007), tomato (Toorop et al., 2000; Wu et al., 2000), coffee (Da Silva et al., 2004), cucumber (Amritphale et al., 2005; Salanenka et al., 2009), and lettuce (Nascimento et al., 2000).

In the mature seed, cells of the micropylar endosperm store hemicelluloses in cell walls, and proteins, lipids and minerals in the cytoplasm. Hemicelluloses strengthen and harden the tissue, and germination is only possible after this tissue has been sufficiently weakened (i.e., hydrolyzed) and the radicle can overcome its resistance (Psaras et al., 1981; Psaras and Georghiou, 1983; Watkins and Cantliffe, 1983; Groot and Karssen, 1987; Sánchez et al., 1990). In quinoa, control of radicle protrusion during germination is mediated, at least in part, by micropylar endosperm weakening, but its emergence occurs via the channel occupied by suspensor remains (unpublished observations).

On the other hand, endosperm weakening, as well as radicle growth potential, are known to be regulated by plant hormones (Linkies et al., 2010): abscisic acid (ABA) inhibits endosperm weakening while gibberellins (GA) act as its antagonists in a complex network integrating environmental signals such as light, temperature, water availability, and nutrient status (Kucera et al., 2005); ethylene and brassinosteroids also counteract ABA, but their effects on endosperm weakening are unknown.

In grasses, there is no micropylar endosperm, and the coleorhiza plays a role in protecting emerging roots during germination (Sargent and Osborne, 1980). According to Nishimura (1922), Howarth (1927), Walne et al. (1975), and Debaene-Gill et al. (1994), the coleorhizae also act in water and nutrient uptake, as a water reserve during dehydration and as a storage tissue. Barrero et al. (2009) propose a new role for the coleorhiza, i.e., to regulate germination in dormant seeds. Recent studies have shown that ABA 8-hydroxylase gene expression is strong and uniform in barley coleorhizae, thus suggesting a key role for this tissue in dormancy control, equivalent to that of the micropylar endosperm (Millar et al., 2006). In fact, several alternative catabolic pathways exist for the inactivation of ABA (Zhou et al., 2004; Nambara and Marion-Poll, 2005), but the reaction catalyzed by ABA 8′-hydroxlyase is considered to be predominant in ABA catabolism (Nambara and Marion-Poll, 2005). ABA 8′-hydroxlyase gene expression occurs in the coleorhiza and does not occur anywhere else in the barley embryo (Millar et al., 2006).

## SEED STORAGE RESERVES

### STARCH

In quinoa perisperm, starch accumulates forming compound and simple grains (Prego et al., 1998). In cereal starchy endosperm, compound grains have been reported in barley, rice, and wheat (Matsushima et al., 2010; Yun and Kawagoe, 2010). In wheat and barley, two types of starch grains are present: the large and lenticular A-type, which contains higher amylose concentrations, and the small spherical or ovoide B-type (Peng et al., 1999). In maize, according to Katz et al. (1993), starch grains are simple grains, but during development, Shannon et al. (1998) report the presence of both simple (larger) and compound (smaller) amyloplasts.

**FIGURE 7:**
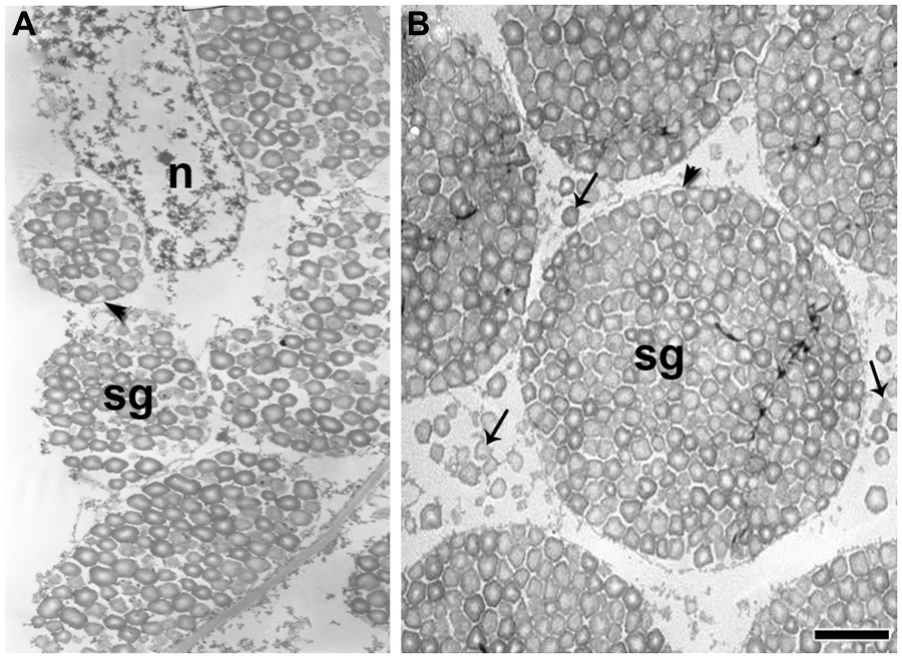
**Ultrathin sections of the quinoa perisperm in two subsequent developmental stages.** Grains were fixed and embedded as described in **Figure 2**. Ultra-thin sections were mounted on grids coated with Formvar and stained with uranyl acetate followed by lead citrate.**(A)** Numerous simple starch grains are being packed inside amyloplasts (arrowhead) originating compound grains; *n*, nucleus; *sg*, starch grains.**(B)** Simple starch grains (arrows) and compound starch grains (arrowhead). Bar: 1 μm.

In quinoa, compound starch grains originate inside the amyloplasts by aggregation of single grains (**Figure 7A**). At the end of development, single grains are deposited in the extraplastidial space (**Figure 7B**). TEM images suggest that they are cytosolic in origin, but further studies are needed, considering that, to our knowledge, the formation of starch grains in the cytosol has not been previously reported. In rice endosperm, compound grains are generated by divisions of amyloplasts, which occur simultaneously at multiples constriction sites, and small amyloplasts bud from their surface (Yun and Kawagoe, 2009). More recently, Yun and Kawagoe (2010) demonstrated that a septum-like structure containing inner envelope membrane divides granules in the amyloplast and that proteins of organelle division, (including FtZ, Min, ARC5, and PDV2) play roles not only in amyloplast division but also in compound granule synthesis (Yun and Kawagoe, 2009, 2010). These results strongly suggest that amyloplast division and compound granule synthesis in rice are closely related. This needs to be investigated further, since starch biosynthesis is an essential function in plant metabolism and is highly conserved throughout the plant kingdom.

Starch is synthesized from sucrose as a result of the combined action of four distinct enzymes: ADP-Glc pyrophosphorylase (AGPase), starch synthase (SS), starch-branching (BE), and starch-debranching (DBE) enzymes (Hannah, 2007). The complement of starch biosynthetic enzymes is well conserved between plastids of tissues that make different types of starches, i.e., transitory starch (made in chloroplasts) and storage starch (made in amyloplasts; Tetlow, 2010; for review see Geigenberger, 2011). In dicot storage tissue, the synthesis of ADP-glucose by the enzyme ADP-glucose AGPase occurs entirely within plastids, as reported in potato tuber (Sweetlove et al., 1996), pea embryo, and root (Denyer and Smith, 1988; Smith, 1988). In contrast, in monocots/cereals, i.e., in maize (Denyer et al., 1996; Huang et al., 2014), barley (Thorbjørnsen et al., 1996), rice (Sikka et al., 2001), and wheat (Tetlow et al., 2003), there is evidence indicating the presence of AGPase enzymes corresponding to plastidial and cytosolic isoforms (for reviews see Hannah and James, 2008; Comparot-Moss and Denyer, 2009; Geigenberger, 2011). Furthermore, whereas SS, BE, and DBE enzymes are found within amyloplasts, AGPase activity (which represents the rate-limiting step in starch biosynthesis) is confined almost exclusively to the cytosol. According to Beckles et al. (2001), the cytosolic localization of AGP in cereal endosperm may have functional significance for partitioning large amounts of carbon into starch when sucrose is plentiful.

In quinoa, it has yet to be determined if the synthesis of ADP-glucose by the enzyme ADP-glucose AGPase occurs entirely in plastids, as in the rest of dicotyledons studied to date. However, in advanced stages of quinoa seed development, once compound starch grain formation is complete, simple starch grains do form filling the space between amyloplasts (López-Fernández and Maldonado, 2013a; **Figure 7B**).

In cereal starchy endosperm dead cells, protein bodies, which mainly store prolamins, are present in the extraplastidial space. Conversely, in dead cells of quinoa perisperm, starch is the only storage reserve.

### PROTEINS, MINERALS, AND LIPIDS

In quinoa, embryo and micropylar endosperm cells store proteins and lipids in the form of protein storage vacuoles (PSVs) and lipid bodies (Prego et al., 1998; Carjuzaá et al., 2008; López-Fernández and Maldonado, 2013b), respectively. PSVs contain one or more phytin crystals in the proteinaceous matrix and proplastids contain clusters of particles of phytoferritin (Prego et al., 1998). Brinegar and Goundan (1993) describe the protein composition of quinoa seeds and report an 11S-type globulin.

A 2S cysteine-rich globulin (8–9 kDa) is also described for quinoa seeds by Brinegar et al. (1996). More recently, Castellión et al. (2008) confirm these results. Likewise, Balzotti et al. (2008) report the genomic and cDNA sequences for two 11S genes from the quinoa genome; in addition, on the basis of comparison with orthologous 11S sequences from other species, they describe the characteristics of the genes and their encoded proteins.

**FIGURE 8:**
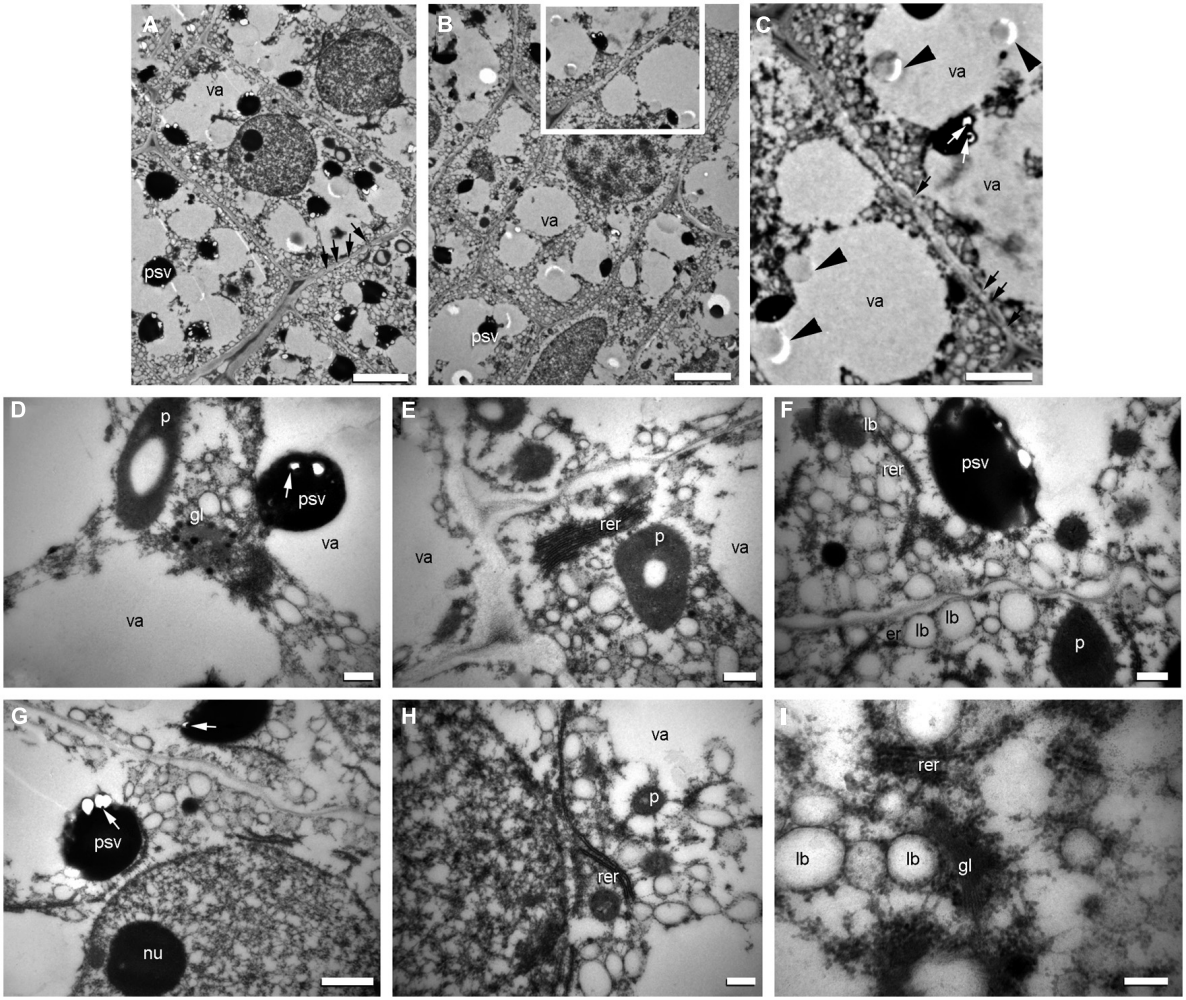
**Origin of protein storage vacuoles (PSVs) and lipid bodies in quinoa embryo.** Excised embryos (torpedo stage) were processed as indicated in **Figure 7**. **(A)** Cells from the ground meristem. **(B)** Cells from the procambium. **(C)** detail of **(B)**. **(D–I)** Details of cytoplasm and organelles in cells from the ground meristem. In the different images, intense biosynthetic activity in cytoplasm can be inferred by the presence of abundant cisternae of rough endoplasmic reticulum (*rer*), numerous *PSVs* originating from the vacuoles (*va*); globoids (white arrows) can be seen inside PSV; the empy areas inside PSV contained globoid crystals before they were dissolved during treatment of tissue fixation; numerous lipid bodies (*lb*) associated with endoplasmic reticulum; frequent dictiosomes, or Golgi (*gl*) and circular vesicles with electronically dense content in proximity to the dictiosomes (see **D**); nuclei with one or more nucleoli (*nu*). Arrowheads indicate the presence in the vacuole of the precursor salts of the globoids; white arrows indicate globoids; black arrows indicate plasmodesmata. *p*, plastid. Bars: **A–B**, 2 μm; **C**, 1.5 μm; **D–I**, 0.5 μm.

During development, proteins are transported from the lumen of the rough endoplasmic reticulum (RER) to the vacuole through the Golgi apparatus and PSVs are formed when vacuoles fragment (**Figure 8**). For each PSV, several electron-dense globoid crystals are formed (**Figure 8**). Before globoid formation, vesicles of phytic acid can be observed as bubbles concentrated on the outside of the PSV (**Figures 8A,B**); there, phytic acid associates with ions to form electron-dense globoids of phytin. To our knowledge, the formation of globoid crystals in the PSV had not been previously photo-documented in angiosperm seeds. Energy dispersive X-ray (EDX) analysis of globoid crystals reveals the presence of P, K, and Mg (Prego et al., 1998).

In cereals, globulins are synthesized in both the aleurone layer and starchy endosperm, and prolamins synthesized within the starchy endosperm rather than in the aleurone layer (Kriz and Schwartz, 1986; Kriz, 1989; Kriz and Wallace, 1991; for review see Shewry and Halford, 2002). On the other hand, protein body formation is tissue specific: in the aleurone layer, the pathways for all globulins and some albumins are similar to that described for quinoa, i.e., protein vesicles are transported from the lumen of the RER to the vacuole by way of the Golgi apparatus, and PSVs are formed by the subsequent fragmentation of the vacuole. Prolamins accumulate in protein bodies inside the endoplasmic reticulum (ER) of starchy endosperm cells, excluding the Golgi and vacuoles (Levanony et al., 1992; Rechinger et al., 1993; Reyes et al., 2011). Crystal globoids, which are included in the PSVs, are detected in the scutellum and aleurone layer but not in the starchy endosperm (Tanaka et al., 1973; Ogawa et al., 1977).

Lipid bodies are found in the quinoa embryo and micropylar endosperm, as well as in grass aleurone and embryo tissues. Lipid bodies originate in the ER (Schwarzenbach, 1971; Wanner et al., 1981; Hsieh and Huang, 2004). Lipids (triacylglycerides) accumulate between the bilayer leaflets at ER specific sites and, when they reach a certain size, bud off from the ER. Oleosin proteins are small (∼15–30 kDa) and abundant proteins in the seeds of plants that bind to the surface of lipid bodies (Chapman et al., 2012). The synthesis and incorporation of oleosins occurs as triacylglycerides are deposited within the lipid bodies (Hsieh and Huang, 2007). During quinoa seed development, lipid bodies originate from the ER at the same time as the PSVs (**Figure 8**).

### ENDOPOLIPLODY AND PROGRAMMED CELL DEATH IN STORAGE SEED TISSUES: QUINOA PERISPERM vs. CEREAL STARCHY ENDOSPERM

Quinoa perisperm consists of uniform, non-living, thin-walled cells full of starch grains. In grass starchy endosperm cells are full of starch grains, but also contain prolamin protein bodies. Thus, the two storage tissues are similar in terms of general characteristics and function, although genetically different.

During perisperm and endosperm development, two important aspects of both quinoa perisperm and cereal starchy endosperm are endoreduplication (for a review see Larkins et al., 2001) and PCD (Young et al., 1997). In both tissues, cell death and dismantling are temporally separated, a process that can take years depending on when germination takes place (Young et al., 1997; Young and Gallie, 1999, 2000; López-Fernández and Maldonado, 2013a). In both cases, cell death occurs during seed formation and is not due to a complete process of autophagy since the cell remains intact until germination, when the starch reserves are mobilized and the dead tissue is finally dismantled. According to van Doorn and Woltering (2005), this type of cellular death does not seem to be autophagic in the strict sense of the term. In a more recent classification, based on morphological criteria, van Doorn et al. (2011) recognize two major classes of cell death that occur in plant tissues: vacuolar cell death and necrosis. However, PCD in grass starchy endosperm (Van Doorn, 2011; van Doorn et al., 2011) and quinoa perisperm, does not strictly fall into these two categories and are classified as separate modalities (van Doorn et al., 2011).

## CONCLUDING REMARKS

In spite of their different origins, quinoa perisperm and grass starchy endosperm exhibit similar developmental programs and functional fates, i.e., endoreduplication, starch accumulation, and programmed cell death. Given the conservation of this seed developmental trajectory in quinoa and cereals, we infer the existence of convergent evolution in these two phylogenetically distant taxa.

The micropylar endosperm of quinoa and the coleorhiza of cereals serve the same role during germination: both tissues store reserves, protecting the root apical meristem in the quiescent seed and control dormancy during germination.

Because of the supposedly independent origin of monocotyledons and dicotyledons, efforts to solve questions related to seed evolution and particularly that of what determines the fate of storage tissues should be furthered for both grasses and quinoa. Hence, the present study may constitute a contribution toward a more complete understanding of seed biology and thus may provide support for broader phylogenetic studies.

## Conflict of Interest Statement

The authors declare that the research was conducted in the absence of any commercial or financial relationships that could be construed as a potential conflict of interest.
